# Synergistic Inhibition of Mammary Carcinoma Transplants in A-Strain Mice by Antitumour Globulin And C. parvum

**DOI:** 10.1038/bjc.1971.74

**Published:** 1971-09

**Authors:** M. F. A. Woodruff, M. P. Inchley

## Abstract

It has been confirmed that the growth of intrastrain transplants of a mammary carcinoma in A/HeJ mice is inhibited to a moderate extent by giving the prospective recipient an intravenous injection of killed *C. parvum* 2 days before tumour inoculation. Intraperitoneal injection of *C. parvum* gave similar results but subcutaneous injection was less effective. Incubation of tumour cells with heterospecific antitumour globulin (ATG) in the absence of complement before inoculation sometimes but not always resulted in moderate inhibition of tumour growth. When pre-incubation of tumour cells with ATG and treatment of the host with *C. parvum* were combined the inhibitory effect was much greater than that produced by either procedure alone, and was roughly equivalent to reducing the dose of viable cells in control animals by a factor of 100. Since A/HeJ mice lack the fifth component of complement (C′5) it was expected that this serum would be ineffective as a source of C′ in *in vitro* cytotoxic tests but effective in opsonisation tests. This has been confirmed. The possible significance of this finding in relation to the synergistic effect of ATG and *C. parvum* is discussed.


					
584

SYNERGISTIC INHIBITION OF MAMMARY CARCINOMA TRANS-

PLANTS IN A-STRAIN MICE BY ANTITUMOUR GLOBULIN
AND C. PARVUM

M. F. A. WOODRUFF AXD M. P. INCHLEY

From the Department of Surgery, University of Edinburgh

Received for publication July 27, 1971

SUMMARY.-It has been confirmed that the growth of intrastrain transplants
of a mammary carcinoma in A/HeJ mice is inhibited to a moderate extent by
giving the prospective recipient an intravenous injection of killed C. parvum
2 days before tumour inoculation. Intraperitoneal injection of C. parvum gave
similar results but subcutaneous injection was less effective. Incubation of
tumour cells with heterospecific antitumour globulin (ATG) in the absence of
complement before inoculation sometimes but not always resulted in moderate
inhibition of tumour growth. When pre-incubation of tumour cells with ATG
and treatment of the host with C. parvum were combined the inhibitory effect
was much greater than that produced by either procedure alone, and was
roughly equivalent to reducing the dose of viable cells in control animals by a
factor of 100. Since A/HeJ mice lack the fifth component of complement
(C'5) it was expected that this serum would be ineffective as a source of C' in
in vitro cytotoxic tests but effective in opsonisation tests. This has been
confirmed. The possible significance of this finding in relation to the synergistic
effect of ATG and C. parvum is discussed.

As already reported (Woodruff and Smith, 1970), the growth of isogeneic
mammary carcinoma transplants in A-strain mice may be inhibited by treating
the recipient with heterospecific antitumour globulin (ATG), but the degree of
inhibition is variable and never very marked. It was anticipated that pre-
incubation of tumour cells with ATG in the absence of complement (C') before
inoculation would have a more decisive effect. As reported below, however, we
have found that, while tumour growth may be inhibited to a modest extent
following this procedure, it may, on the other hand, be unaffected or even faci-
litated.

It was reported by Cinader, Dubiski and Wardlaw (1964) that A-strain mice,
including in particular mice of the substrain A/HeJ which we have used exten-
sively, lack a component of the complement system which has been subsequently
identified by Nilsson and Miiller-Eberhard (1967) as C'5. This is consistent with
our own previously unreported finding that, despite precautions to avoid inactiva-
tion, including collection over ice (Borsos and Cooper, 1961), A/HeJ serum (unlike
that of CBA and C57BI mice) is completely ineffective when used as a substitute
for guinea pig C' in titrating haemolytic and cytotoxic antibody, whereas it is
effective in opsonisation tests. Such inhibition of tumour growth as occurs in
consequence of treatment of either the tumour cells preceding inoculation or the
recipient with ATG, therefore, cannot be attributed to C'-dependent lysis, and the
most likely explanation of the observations cited would seem to be that ATG

INHIBITION OF MAMMARY CARCINOMA TRANSPLANTS IN MICE

585

functions as an opsonin, and that its effectiveness depends on the level of macro-
phage activity in the host.

If this hypothesis is correct, procedures which stimulate phagocytosis by
macrophages might be expected to potentiate the tumour-inhibiting effect of
ATG.

One such procedure is administration of a killed culture of C. parvum (Halpern,
Prevot, Biozzi, Stiffel, Mouton, Morard, Bouthillier and Decreusefond, 1963). It
has already been reported from this laboratory that intravenous injection of this
material, either 2 days before or 8-12 days after subcutaneous inoculation of
viable mammary carcinoma cells in A-strain mice, significantly delays growth of
the tumour (Woodruff and Boak, 1966; Smith and Woodruff, 1968). The present
experiments were therefore designed to test the prediction that treatment of the
tumour cells with ATG and of the host with C. parvum would combine sy-ner-
gistically, so that the degree of tumour inhibition resulting from the two pro-
cedures together would exceed that expected from simple summation of their
individual effects. As a preliminary we have tested two more strains of C. parvum,
and compared the effect of different routes and schedules of immunization.

MATERIALS AND METHODS

Three experiments were performed. The protocols are shown in Tables I-III.
First experiment.-This was designed to assess the effect of a single subcuta-
neous (s.c.), intraperitoneal (i.p.) or intravenous (i.v.) injection of C. parvum on
the growth of a subcutaneous intrastrain transplant of a mouse mammary carci-
noma. The C. parvum was given 2 days before tumour transplantation. This
particular time interval was chosen in the light of previously reported experiments
with other strains of C. parvum (Woodruff and Boak, 1966; Smith and Woodruff,
1968), and a preliminary trial with the strains used in the present investigation,
which showed that injection 7 days preceding tumour transplantation had
relatively little effect.

Second experiment.-This was designed with two objects in mind. The first
was to extend the scope of the previous experiment (a) by comparing the effect of
two i.p. doses of C. parvum, given on Days -2 and +3 with a single dose on Day
-2 (reckoning the day of tumour transplantation as Day 0), and (b) by studying
the effect of a single dose of C. parvum 2 days before excision of an established
tumour transplant and re-inoculation of a measured number of viable cells. The
second object was to determine the effect of incubating tumour cells with ATG
before injecting them into untreated mice or mice which had been given a single
i.p. dose of C. parvum 2 days previously.

Third experiment.-The third experiment was designed to provide further
information concerning the separate and combined effects of (a) incubating tumour
cells with ATG before transplantation, and (b) treating the prospective recipient
with C. parvum.

Mice.-The mice were adult (18-26 g.) females of strain A/HeJ, obtained from
the Jackson Memorial Laboratories, Bar Harbour, Maine, U.S.A.

Tumour transplantation.-Transplantation was performed by s.e. injection of a
tumour cell suspension prepared with pronase, as described by Wooclruff and
Boak (1966), from a second generation transplant of a mammary carcinoma which
had arisen spontaneously in ainL old A/HeJ female. The proportion of viable cells
determined by a dye-exclusion test with trypan blue, ranged from 75 to 90 per

49

586

M. F. A. WOODRUFF AND M. P. INCHLEY

cent. The tumour dose was expressed as the absolute number of viable tumour
cells injected.

The animals were examined thrice weekly. When a tumour became palpable
its mean diameter was determined with callipers.

C. parvum.-Formalin-killed cultures of two different strains of C. parvum
were used. One preparation was obtained from the Wellcome Foundation
(Batch EZ174) by courtesy of Dr. J. Cameron; the other (Reticulostimuline) from
the Pasteur Institute, Paris, by courtesy of Professor Marcel Raynaud. We have
designated these W2 and P2 respectively to distinguish them from other pre-
parations obtained previously from the same two sources. The nitrogen content,
by microKjeldahl analysis, was 0-96 mg./ml. for W2 and 1-18 mg./ml. for P2.
Both preparations were given in a dosage of 0.2 ml. W2 was used in all three
experiments; P2 in the third experiment only.

Preparation and use of ATO.--Antitumour serum (ATS) was prepared by
immunizing rabbits with a mixture of tumour cells prepared with pronase from
intrastrain transplants of several tumours of the type described above, according
to the following schedule:

Day 0 loo X 106cells in Freund's complete adjuvant distributed between the

four footpads, and 400 x 106 cells in physiological saline i.p.
Day 28 500 x 106 cells in physiological saline i.p.
Day 35 500 x 106 cells in physiological saline i.p.

The rabbits were bled (50 ml.) from an ear vein on Day 45 and exsanguinated by
cardiac puncture on Day 46. The blood was allowed to clot, and the serum was
separated, pooled and inactivated by heating for 30 minutes at 56' C.

ATG was prepared from ATS by two precipitations with an equal volume of
32 per cent sodium sulphate, followed by batch chromatography on Whatman
DE II DEAE =cellulose using a pH 6 - 5 0.02 m phosphate buffer, as described by
Anderson, James and Woodruff (1967). Immunoelectrophoresis revealed only
traces of impurities with an electrophoretic mobility greater than the IgG.

Different batches of ATG were used in the second and third experiments, but
they both had an in vitro cytotoxic titre-1 of 128.

Pre-incubation of tumour cells.-2-107cells were incubated in 2-6 ml. Dulbecco's
solution containing 6-5 mg. ATG at 37' C. in a water bath. As a rule no C' was
included, but as an additional control in the third experiment one group of mice
(group 7) were given cells which had been pre-incubated as described above except
that 0-65 ml. undiluted guinea pig serum had been added as a source of C'.

RESULTS

The results are summarised in the tables and figures. As reported previously
(Woodruff and Boak, 1966), after a lag period during which no tumour was
palpable, there was typically a period of some weeks during which tumour dia-
meters increased in an approximately linear manner with time. Where necessary
we have compared the mean diameter of tumours in treated and control animals
at a time when the latter were at or a little beyond the mid-point of this phase by
a t-test, using either the standard test or, when the variances in the groups com-
pared were significantly different, Bailey's (1959) modification. Sometimes
however the difference in tumour growth in different groups was obvious on
inspection and statistical analysis was not required.

INHIBITION OF MAMMARY CARCINOMA TRANSPLANTS IN MICE

20-
18-
E

E16-

14-                 /X
w

w 12-
7-

< 10-

8-
D
0

m 6

D

1-- 4-
z

w 2-
7-

587

20             30              40
DAYS AFTER INOCULATION WITH TUMOUR CELLS

FiG. l.-First experiment. Effect on tumour growth of a single i.v., i.p. or s.c. injection of

C. parvum.

x ------------ xNo treatment.
x ?  ?   x  C. parvum i.v.
*?   ?   0  C. parvum i.p.
0 ----------- 0C. parvum s.c.

It seems clear from the first experiment (Table 1; Fig. 1) that tumour growth
was delayed by administration of V. parvum by any of the three routes tested, but
intravenous injection was significantly more effective than subcutaneous. The
difference between intraperitone4l and intravenous injection is not significant.

20-

-7

E 18-
E

16-

LU

U-

i2-

10-                                                     X.-Z

XI

o

6-                                  OX

z  4-                                         x

2-i

20

30            40

DAYS AFTER INOCULATION WITH TUMOUR CELLS

FIG. 2.-Second experiment. Effect of an i.p. injection of C. parvum on the growth of (1) a

primary and (2) a secondary tumour transplant.

x ----------- x Primary transplant. No treatment.
x ?? x Primary transplant. C. parvum.

0 ............ 0 Secondary transplant. No treatment.
0 ?? 0 Secondary transplant. C. parvum.

588

M. F. A. WOODRUFF AND M. P. INCHLEY

18-
.-E 16-

IX
ui 14-
w 12-

<

Ei 10-

8-

0                                                                    Je

6-
D
1--

z   4-

<                            All

w   2-

7-             -

0-           I                I                I                1

24        30               40                50               60

DAYS AFTER INOCULATION WITH TUMOUR CELLS

FIG. 3.-Second experiment. Separate and combined effects on tumour growth of (1) i.p.

injection of the host with C. parvitin (W2) and (2) pre-incubation of tumour cells with ATG
(Batch EM).

x ------------ x No treatment.
x       x     C. parvum.

0 Pre-incubation with ATG.

........... * C. parvitm and pre-inctibation.

70

It seems possible, particularly in view of the large variance in tumour size in the
mice given i.p. C. parvum, that a difference might be demonstrated by using more
animals, but in the light of the findings it seemed reasonable to adopt the i.p.

route in preference to the less convenient i.v. route throughout the rest of the

4

investigation.

20-

18-
E

E 16                               /X

14-

/X/

u-J 12-

2                           x

10-

ce- 8-
D

0                    X/                                   .4le

6 -                                               'Ap-

4-                                          Ae
z

<                                         .'

uj 2 -

20             30              40
DAYS AFTER INOCULATION WITH TUMOUR CELLS

50

FiG. 4.-Third experiment. Influence of cell dose on tumour growth.

X............X106 cells.
* ?? 9 10 5 cells.

0............0104 cells.

INHIBITION OF MAMMARY CARCINOMA TRANSPLANTS IN MICE

589

TABLEI.-First Experiment-see also Fig. I

There were 7 mice in each group. All mice received 107 viable tumour cells by subcutaneous injection

to R. flank on Day 0.

Mean tumour diameter on Day + 25

Observed values    Group mean
Group        Treatment                  MM.              MM.

1     Nil                     13, 14, 16, 17, 10, 14, 15  14-1
2     C. parvum (W2)          12, 11, 11, 11, 9, 12, 11  11.0

0-2 ml. s.e. on Day -2

3     C. parvum (W2)          9, 6, 13, 8, 9, 12, 10    9-6

0-2 ml. i.p. on Day -2

4     C. parvum (W2)          9, 10, 8, 7, 11, 9, 10    9.0

0-2 ml. i.v. on Day -2

Statistical comparison of group means by modified t-test (Bailey, 1959)

Groups I and 2 : d = 3-37 f =  8 P =  0-01

4 and 2 : d = 3-24 f = 10 P = <0- 01

In the second experiment primary transplants to the foot grew more slowly
than those to the flank even though the original cell dose was ten times as great
(Table II Groups 1, 6), but when the foot bearing a primary transplant was
amputated and 105 viable cells were re-inoculated to the flank the secondary
transplants grew even more rapidly than primary flank transplants (Table II
Groups 1, 6; Fig. 2). Injection of C. parvum 2 days prior to the re-inoculation
significantly inhibited the growth of the secondary transplant (Table 11 Groups
6 ? 7; Fig. 2).

In this experiment incubation of tumour cells with ATG before transplantation
did not inhibit, indeed if anything it appeared to facilitate, subsequent tumour
growth in untreated mice, but it did markedly potentiate the effect of treating the
host with C. parvum. This is not apparent 40 days after transplantation but after
56 days the synergistic effect of combining the two procedures is strikingly
demonstrated (Table II Groups 1, 2, 4, 5; Fig. 3).

In the third experiment pre-incubation of the cells with ATG without C',
though it did not kill the cells, significantly inhibited subsequent tumour growth,
and was in effect roughly equivalent to reducing the dose of untreated viable cells
by a factor of 10 (Table III Groups 1, 2, 6; Fig. 4, 5). Pre-incubation with ATG
plus C' (Ta'ble III Group 7), which resulted in virtually 100 per cent cell death as
judged by a dye exclusion test, was followed by growth of a tumour in only one
mouse in the group. In the others small nodules appeared but by Day +58 were
still only just palpable (about 1-2 mm. diameter).

The inhibitory effect of C. parvum alone was confirmed (Table III Groups 4, 5;
Fig. 5, 6). The effect of treating the cells with ATG and the host with C. parvum
(Table III Groups 8, 9; Fig. 5, 6) was even more dramatic than in the previous
experiment and was roughly equivalent to reducing the dose of viable cells in
control animals Table III Group 3; Fig. 4) by a factor of 100. Once again W2
was the more effective of the two preparations of C. parvum which were tested
(Table III Groups 4, 5, 8, 9; Fig. 5, 6).

DISCUSSION

The results confirm in one particular model the hypothesis which the experi-
ments were designed to test, namely, that the antitumour effect of heterospecific

49*

590

M. F. A. WOODRUFF AND M. P. INCHLEY

04 0
-0        , p

0          ko

0 ;4                                       06
C)

(D

m

o
0

Cc 00
;4

4a

m o

03 ;, 0                                 -i

-4    -4

0

k                                       00

aq
4a

00

cq

A

0

k
4a

06

0             r-4

;4    0       C4          ?4

0     t

00

NW

v

4a       04 0                                               P-1

0                   mm Wm

0

f*4 +
0
;4

J

10

0                      00      m

cq

cl?   06 cl?                    0 04

0    4a
F-4

0                      0

e.5 4

6eq R4        R. aq                    .4a

.9  1 -4       I"

+

k bo=

O.-

-4       --I
pq

04

0 >

> 0

4z      4a       0

P-40 0      0 C>

..4 0    k

C?o        -.0 C? o    t. . -d 0                0

-4                m    -4 M Q      ;4

k     0     >                ;>? >

-.4                                     0

0               A,                        o 0

0     0

P;    E                                  -la              0,

?4                  k       k P4    At

0                                          0

0          -.3o             &C
4Q 0              0    4a 0 0     0 0 0       0

O g I            g     I    &-.) "  E -,?  0.
:j                        9 0    i  0

.a +;I        -0 4Q    0          4 a

0

.4a
C*40

0 ONP

04                                               k ;4
O
0
k
0

591

INHIBITION OF MAMMARY CARCINOMA TRANSPLANTS IN MICE

C> L- m

C? G :;

C4

,:;    I
P-4

00

P-4

00    ?4

4

C4    ?4
aq

rz    06

P-4   P-4

tz    C;

0 (D

;>

o

06 O

O -CO3

cq 00

O

0        O lqt C) m

>        co C) O

r-4        0 to

C; ci

4a
CB

t-

CT

P-4

C;

06

P-4

to

11.6

ce
P-4

O-A

00
C;
Iz

e-I

Ca
0
Ca

to
03

0
4-D

(74                      "i

P.,       X

MD

Q     0     0      0     C>

P-4                               't

CD

T$ 0               Itz      -tz

11    (D

.'R   -4

10

4S

ow

00 0)
4

co    t-    00     (m

I;o

1?4
4)
I     4a
t? (E)

,xi, i 1-4

.- aq
- Itz >?,

'"       Cs <
t? 0

. eQg 0

N Q 0

(Z) 0

03

e    4)

%) Pi

I?p

....L
9
w

FE

94
zi
;?q

le.
. ez,

PA;:?

PA
?-l

pq          c

!4
E--i

0

'-) w

0. . . . ai

2 . - . 'o . '.4 ?:

I   P-1 zzz

. . . .

0

m 0 0 " 0

0 O 0 O O

9? P-4 P-4 F--4 F-4

0

O

E--l

m

-4
a)
C)

20-

18-
E

E 16-
1--l-

wx 14-
1--

7-w 12-
a lu-

cx

D  8-
0

D7- 6-

1--

z  4-

w  2-
x

n ?

I       0-

592

M. F. A. WOODRUFF AND M. P. INCHLEY

.IX
x .0,

,x/

x

.IX/

)e
.1

X-- - -_x

-i

10               20               30               40               50

DAYS AFTER INOCULATION WITH TUMOUR CELLS

FIG. 6.-Third experiment. Separate and combined effects on tumour growth of (1) i.p.

injection of the host with C. parvum (W2) and (2) pre-incubation of tumour cells with ATG
(Batch EM).

x ........... x No treatment.
x       x     C. pa-rvum.

0 Pre-incubation with ATG.

............ 0 C. parvum and pre-incubation.

20

18
E

E 16-

.1X
01
w 14-

$-_                               x
w

i2-                         X,

oe
C) 10-

(X                                 X,

D   8                                                  AW
0                      X"

7-                                               .1

D 6-

z 4-
w 2-

0

10               20               30               40'

DAYS AFTER INOCULATION WITH TUMOUR CELLS

FIG. 6.-Third experiment. Separate and combined effects on tumour growth of (1) i.p.

injection of the host with C. parvum (P2) and (2) pre-incubation of tumour cells with ATG
(Batch EM).

x ------------ x No treatment.
x       x     C. parvum.

0 Pre-incubation with ATG.

............ 0 C. parvum and pre-incubation.

INHIBITION OF MAMMARY CARCINOMA TRANSPLANTS IN MICE             593

antibody can be markedly potentiated by a procedure which stimulates phagocytic
activity.

It remains to be seen whether this conclusion is of general validity or whether
the findings were determined by special features of the particular model chosen,
including the type of tumour and the strain of mouse. For example, A/HeJ
mice, being deficient in C'5, are presumably more dependent upon phagocytosis as
a means of immunological defence than mice which are not so deficient, and it is
therefore conceivable that they would show a relatively greater response to stimu-
lation by C. parvum.

To test this we have set up experiments similar to those described with trans-
plants of chemically induced sarcomas in A/HeJ, CBA and C57BL mice. We are
also studying the effect of treating the tumour bearing animals with ATG and
C. parvum instead of treating the tumour before inoculation with ATG and the
animals with C. parvum as in the present investigation. It would seem premature
to consider possible clinical applications until the results of these additional
experiments are available.

We gratefully acknowledge generous gifts of C. parvum preparations from the
Wellcome Foundation (Dr. J. Cameron) and from Professor Marcel Raynaud of
the Pasteur Institute, Paris. We are indebted also to Mrs. Evelyn Pawley for
skilled technical assistance.

The work was supported by a grant from the Medical Research Couricil.

REFERENCES

ANDIMRSON, N. F., JAmims, K. AND WOODRUFF,M. F. A.-(1967) Lancet, i, 1126.

BAILEY, N. T. J.-(1959) 'Statistical Methods in Biology'. London (EngHsh Univer-

sities Press).

BORSOS, T. AND COOPER,M.-(1961) Proc. Soc. exp. Biol. Med., 107, 227.

CINADIMR, B., DuBisKi, S. A-WD WARDLAW, A. C.-(1964) J. exp. Med., 120, 897.

HALPERN, B. N., PREVOT, A.R., Biozzi, G., STIFFEL, C., MOUTON, D., MORARD, J. C.,

BouTHTT,T ER, Y. ANDDECREUSEFOND, C.-(1963) J. Beticulo-endothelial Soc., 1,
77.

NmsSON, U. R. AND WLLER-EBERHARD, H. J.-(1967) J. exp. Med., 125, 1.

SimaTH,LINDSAY H. AND WOODRUFF,M. F. A.-(I 968) Nature, Lond., 219, 197.
WOODRUFF,M. F. A. ANDBoAK, J. L.-(1966) Br. J. Cancer, 20, 345.

WOODRUFF,M. F. A. AND SMITH, LrNDSAY H.-(1970) Nature, Lond., 225, 377.

				


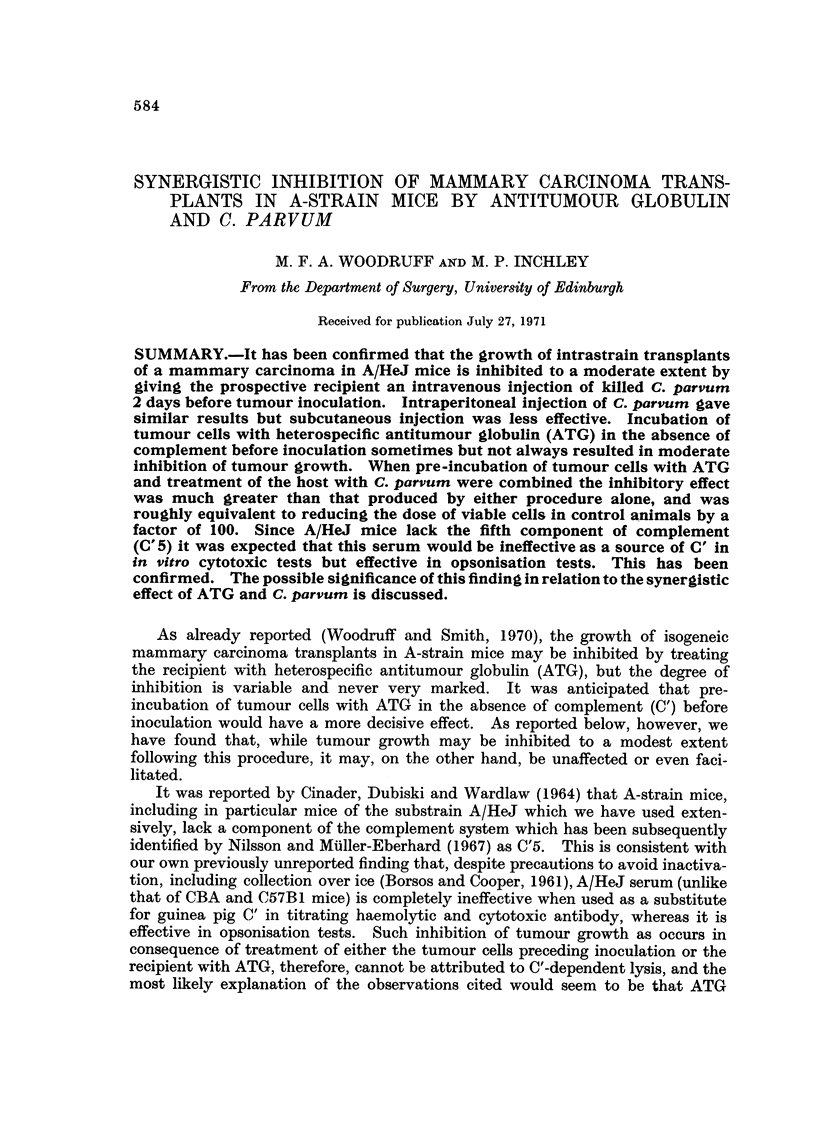

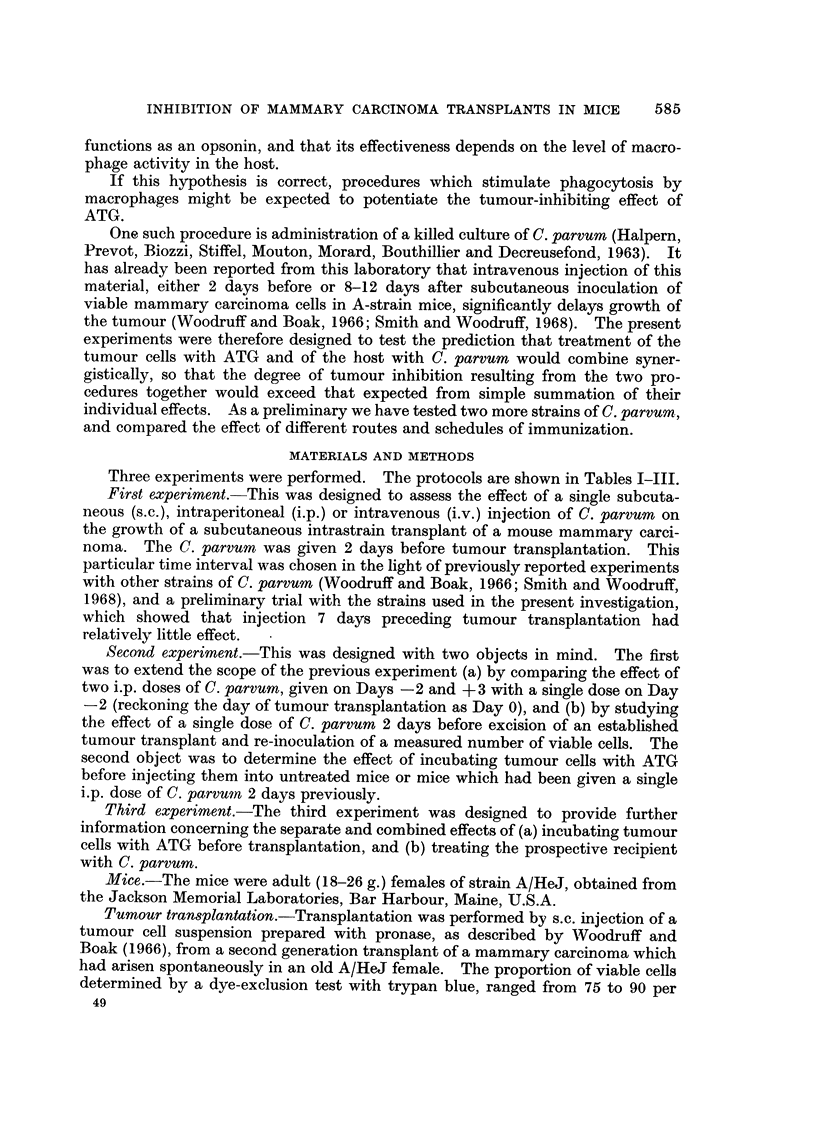

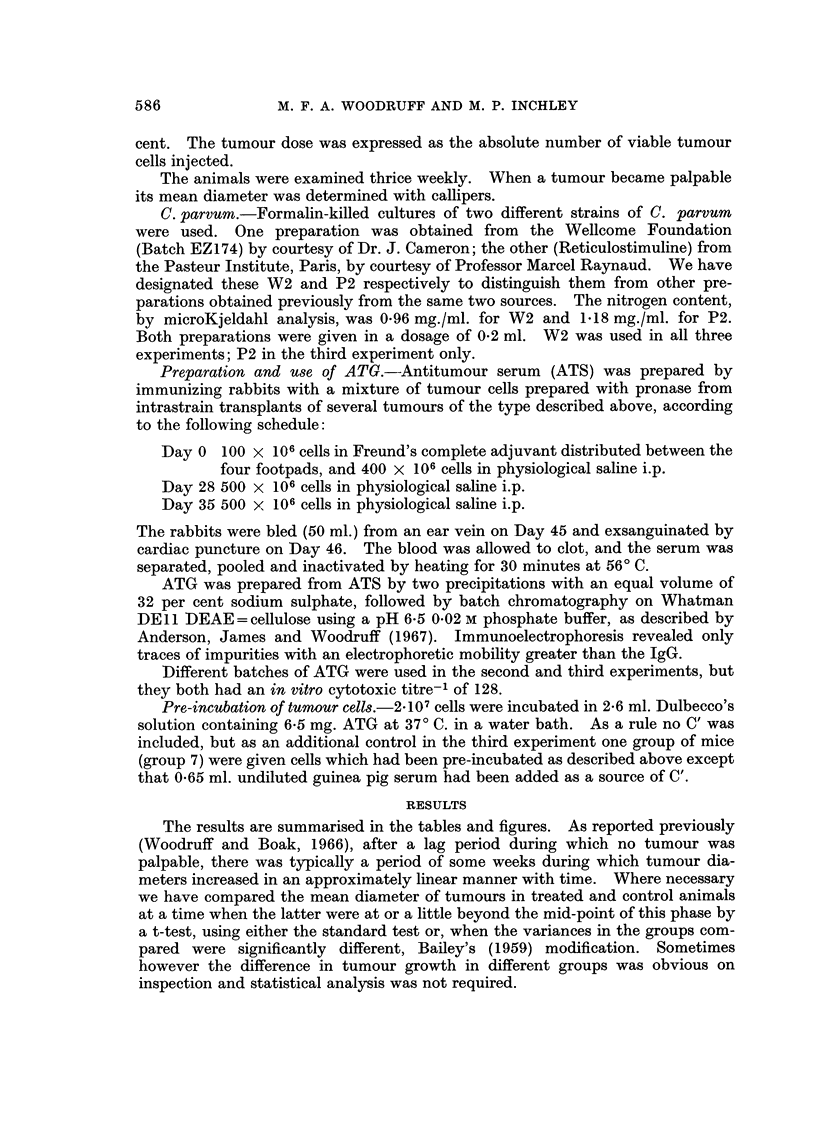

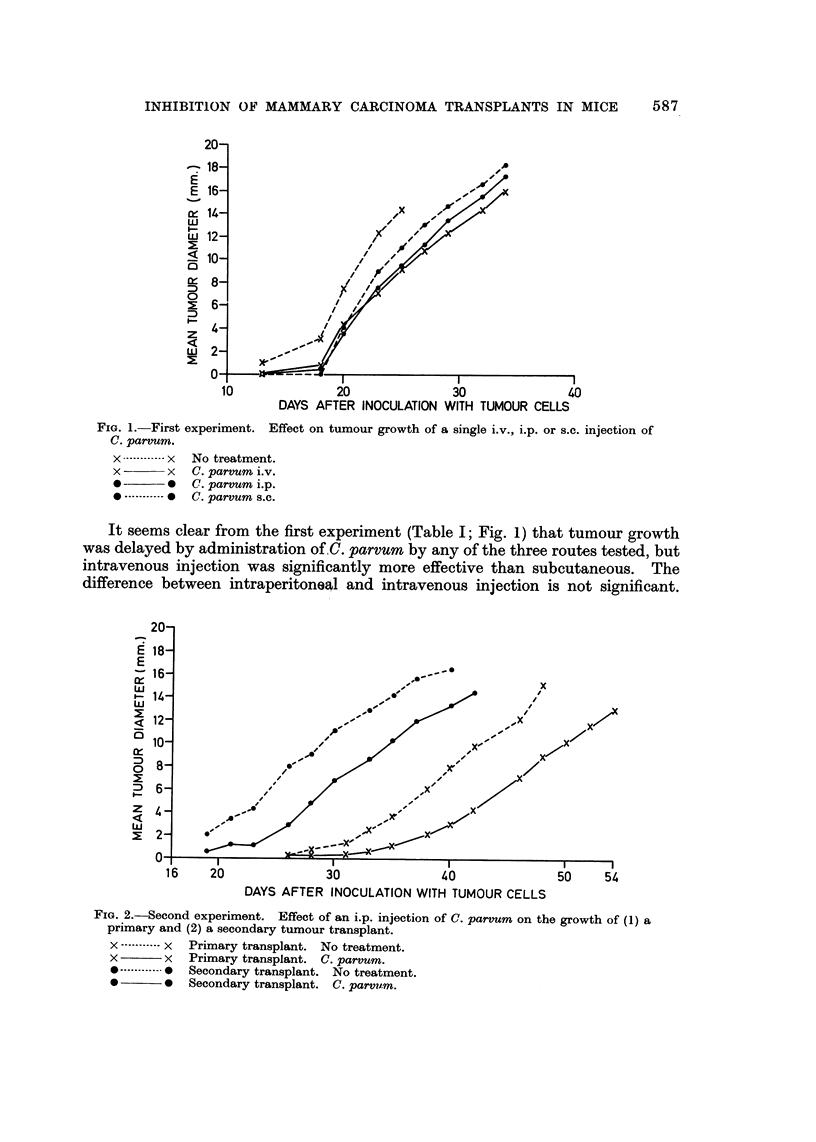

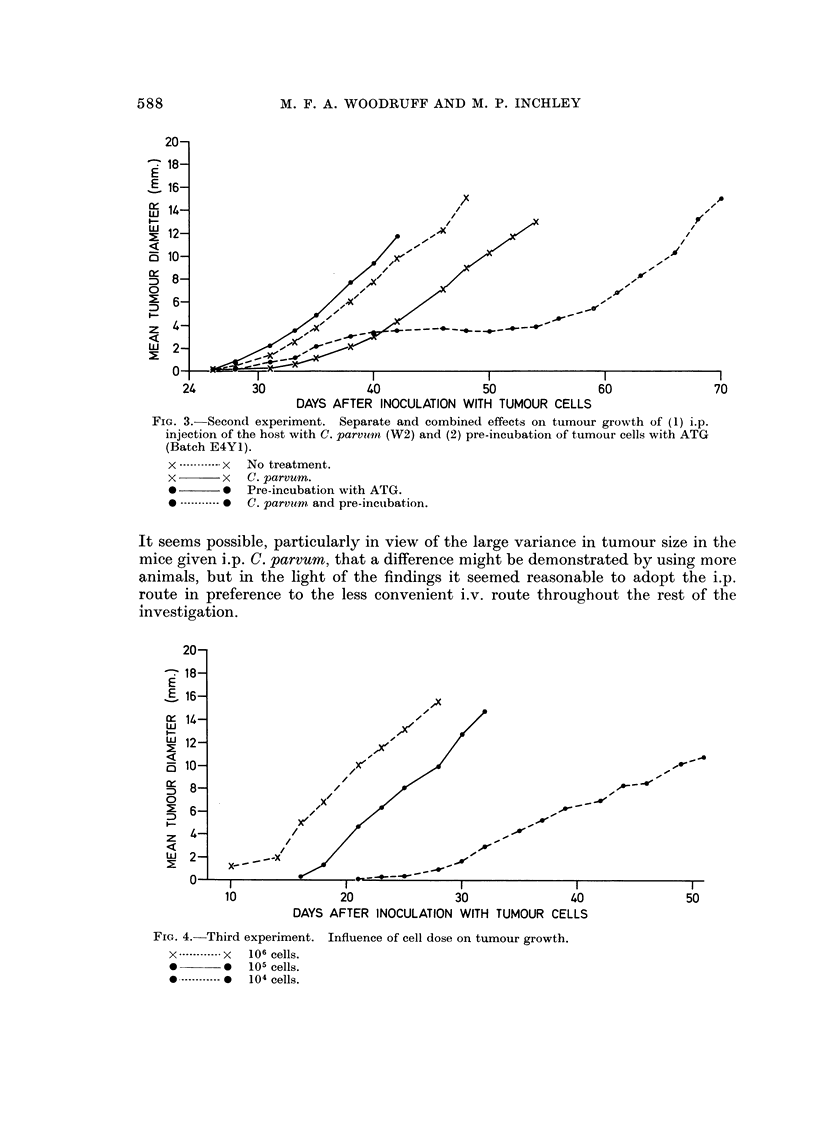

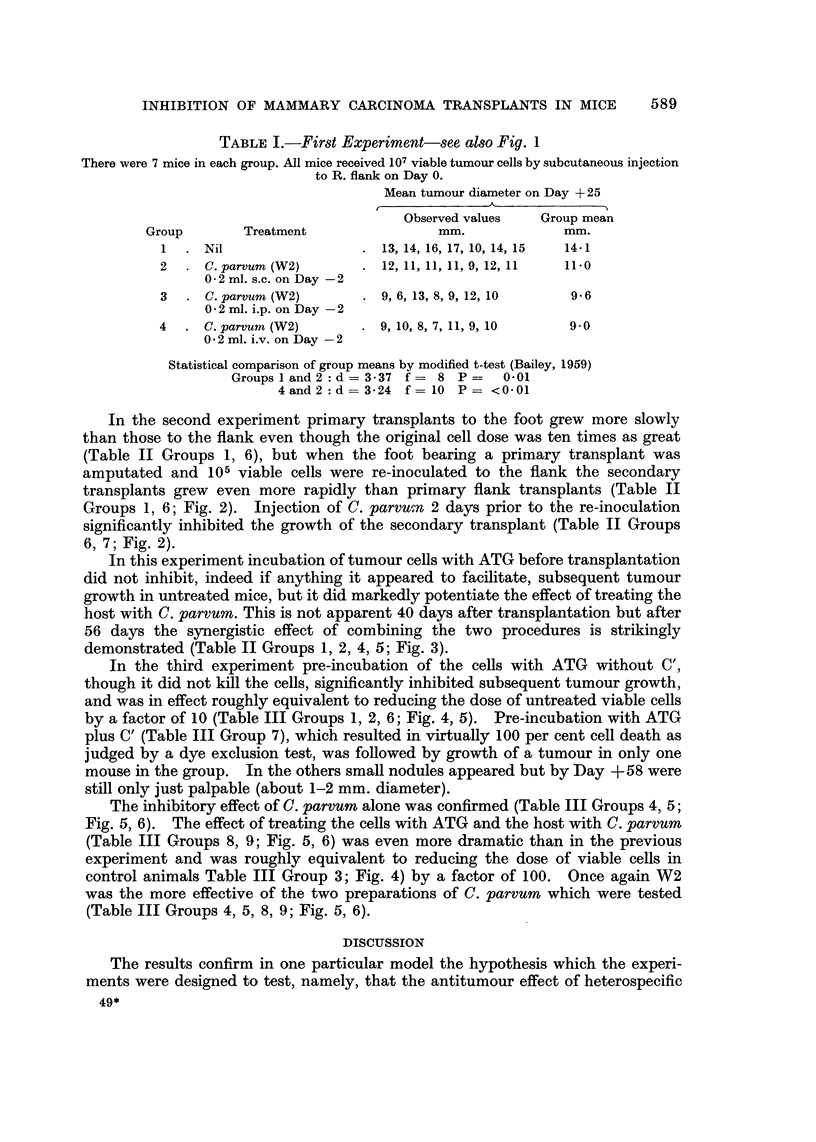

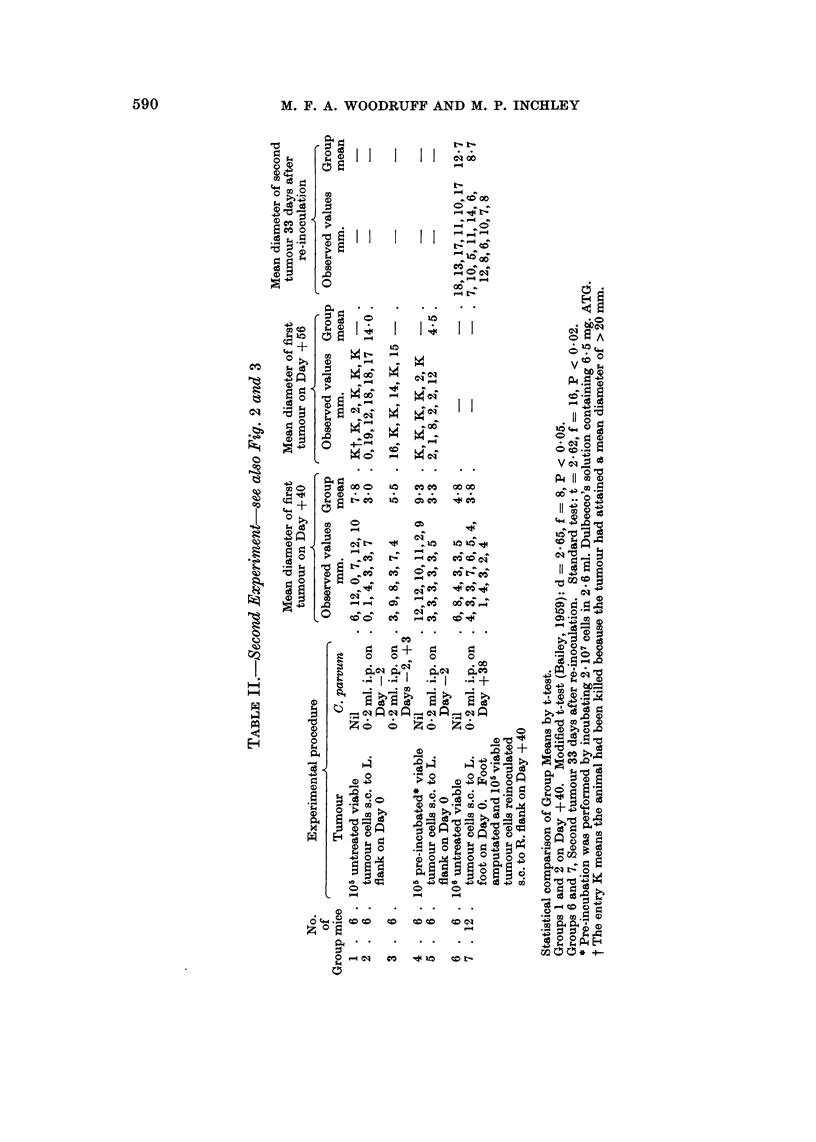

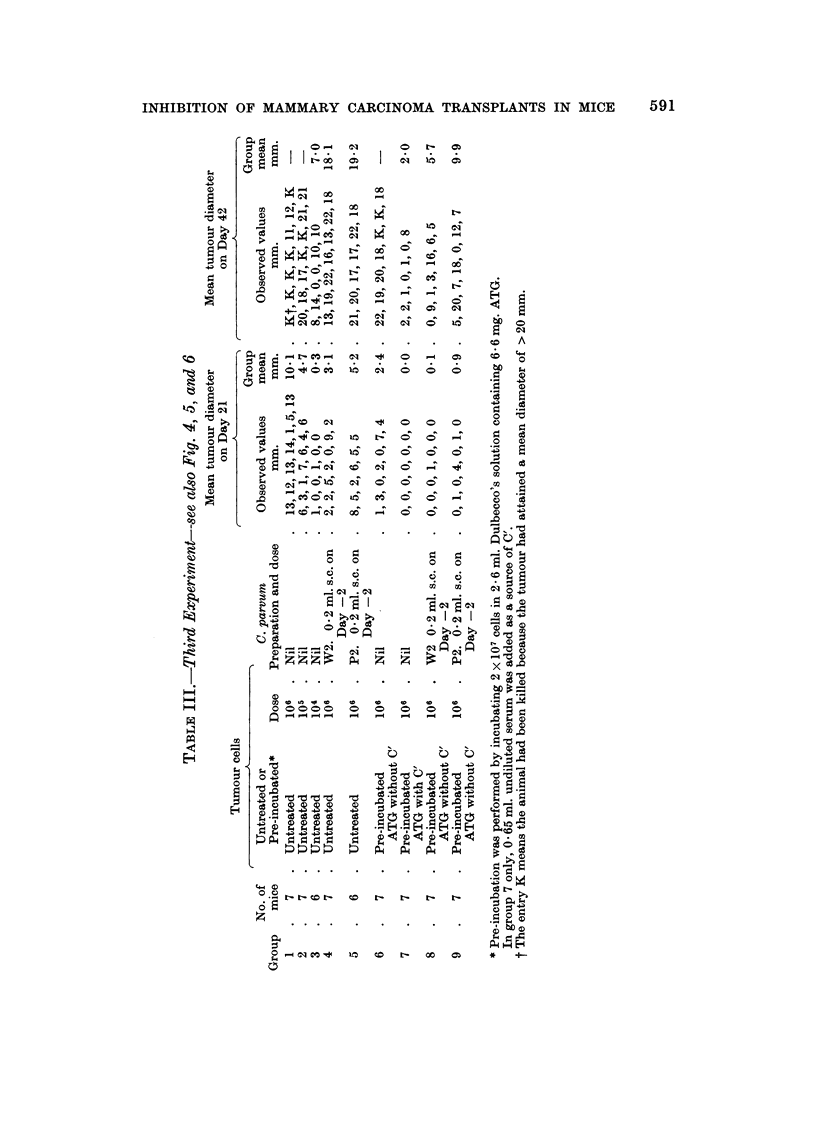

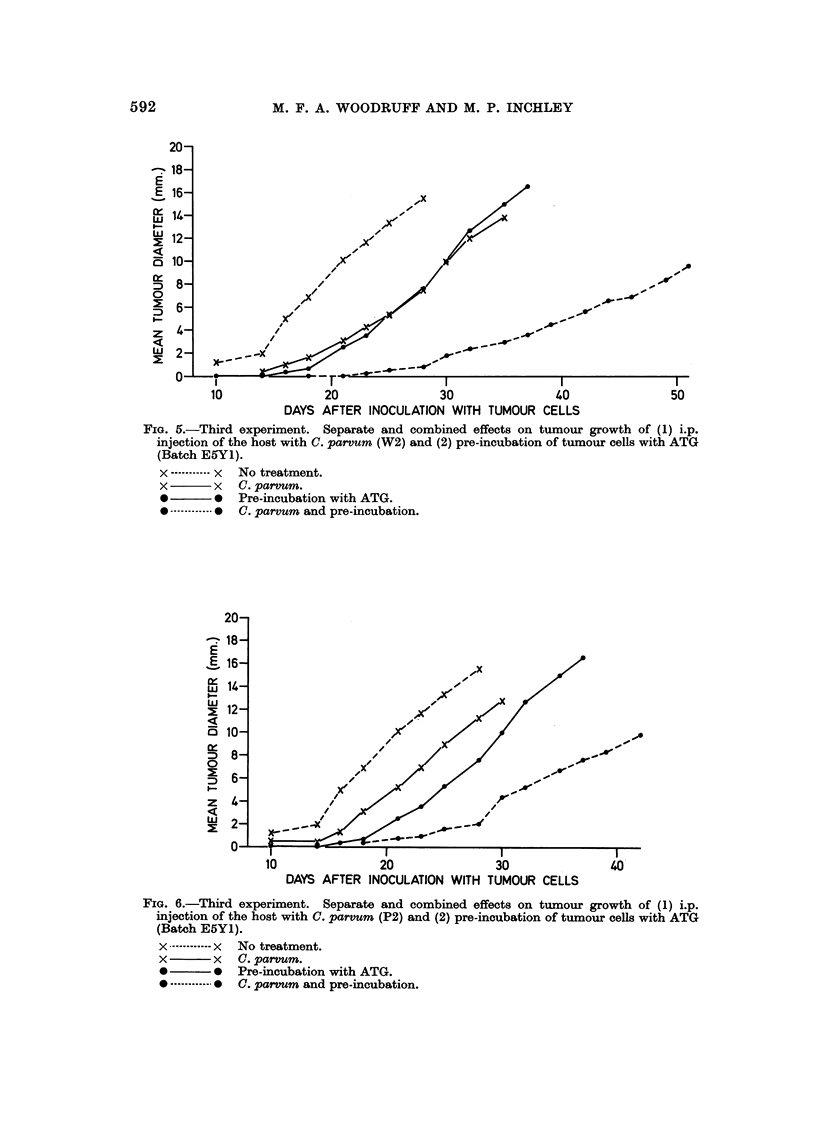

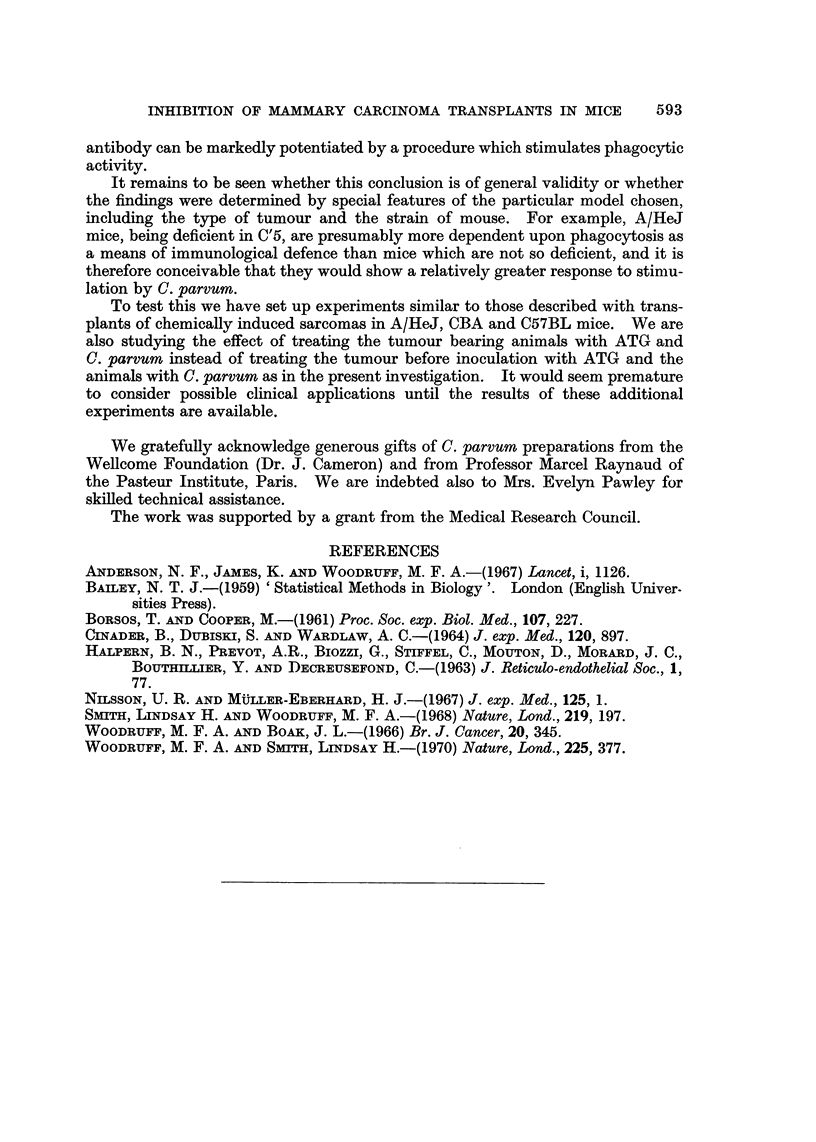

